# Development of a Radiolabeled Folate-Mediated Drug Delivery System for Effective Delivery of Docetaxel

**DOI:** 10.1021/acsomega.3c02656

**Published:** 2023-07-04

**Authors:** Oğuz Çetin, Burcu Güngör, Çiğdem İçhedef, Yasemin Parlak, Elvan Sayıt Bilgin, Funda Üstün, Gülay Durmuş Altun, Yücel Başpınar, Serap Teksöz

**Affiliations:** † Department of Nuclear Applications, Institute of Nuclear Sciences, Ege University, Izmir 35100, Turkey; ‡ Department of Nuclear Medicine, School of Medicine, Celal Bayar University, Manisa 45040, Turkey; § Department of Nuclear Medicine, Faculty of Medicine, Trakya University, Edirne 22030, Turkey; ∥ Department of Pharmaceutical Biotechnology, Faculty of Pharmacy, Ege University, Izmir 35040, Turkey

## Abstract

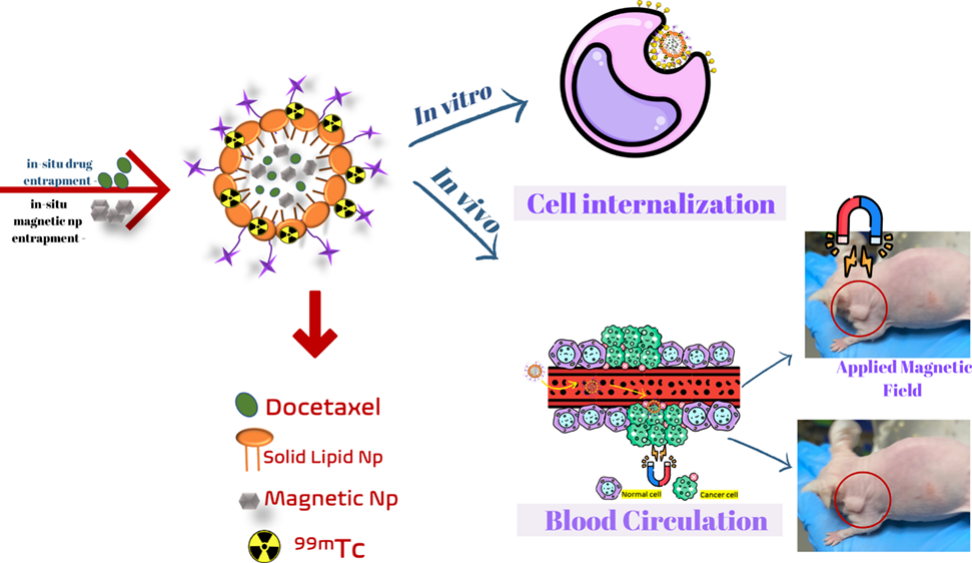

Many preclinical studies are carried out with the aim of developing new formulations for the effective delivery of taxane class drugs, one of the most important anticancer drugs used clinically today. In this study, a radiolabeled folate-mediated solid lipid magnetic nanoparticle (SLMNP) system was developed by loading superparamagnetic iron oxide nanoparticles (MNP) and docetaxel (DTX) into the solid lipid nanoparticles as a drug delivery system that will function both in cancer treatment and diagnosis. For this purpose, first, SLMNP was synthesized by the hot homogenization method, and the surface of the particles was modified with a folate derivative to carry the particles to tissues with folate receptors. The synthesized magnetic solid lipid nanoparticles were loaded with DTX, and then radiolabeling was carried out with technetium-99 m (^99m^Tc-DTX-SLMNP). Structural characteristics of these nanoparticles were determined by characterization methods. According to the TEM images of MNPs, SLN, and SLMNPs, MNPs were observed between 25and 35 nm, SLNs between 400 and 500 nm, and SLMNPs between 350 and 450 nm. The drug entrapment efficiency of SLMNPs loaded with DTX was found to be 19%, and the percentage efficiency of radiolabeling was found to be 98.0 ± 2.0%. The biological behavior of this radiolabeled system was investigated *in vitro* and *in vivo*. Folate receptor-positive SKOV-3 and folate receptor-negative A549 cancer cell lines were studied. The IC_50_ values of DTX-SLMNP in SKOV-3 and A549 cells were 50.21 and 172.27 μM at 48 h, respectively. Gamma camera imaging studies of ^99m^Tc-DTX-SLMNP and magnetically applied ^99m^Tc-DTX-SLMNP compounds were performed on tumor-bearing CD-1 nude mice. The uptake in the folate receptor-positive tumor region was higher than that in the folate receptor negative tumor region. We proposed that the drug delivery system we prepared in this study be evaluated for preclinical studies of new drug carrier formulations of the taxane class of anticancer drugs.

## Introduction

Molecular imaging is a noninvasive medical imaging technique that can provide detailed images and information at molecular and cellular levels. Its importance in clinical applications has been amplified by the recent increase in demand for cancer diagnosis.

This, combined with the use of various molecular imaging techniques with radionuclides, has led to rapid advances in the modern healthcare system.^1,2^

Thanks to various new molecular imaging applications, the pathological development of diseases has been understood, and drug discovery and development have been enabled in the fight against diseases.^2^ However, the primary problem in cancer treatment protocols is nonspecific drug distribution and serious side effects resulting from the rapid clearance of the drug from the circulation. The essential requirement of all anticancer treatments is the delivery of suitable therapeutic agents.

It is well known that nanotechnology is a research area that has great potential and is used in many rapidly expanding sectors by manipulating matter on an atomic or molecular level having new properties.^1−3^ Nanotechnology-based novel carrier systems offer great promise in medicine, especially in the field of cancer, which will revolutionize drug delivery systems, gene therapy, diagnosis, and many research, development, and clinical applications.

Today, the development of nanoparticle-based drug delivery systems has great benefits in biomedical applications for molecular imaging and therapeutic systems.^1^ The nanoparticles which are used for drug delivery purposes consist of polymers (polymeric nanoparticles, micelles, or dendrimers), lipids (liposomes), viruses (viral nanoparticles), organometallic compounds (nanotubes), and inorganic nanoparticles (fullerenes, carbon nanotubes, quantum dots, and magnetic nanoparticles), as well as polymer proteins. The physical and chemical properties of nanoparticles have an important role in determining the particle–cell interaction, cellular escape mechanism, biodistribution, and pharmacokinetic and optical properties.^4^ The use of nanostructured systems in the diagnosis and treatment of cancer not only helps to deliver chemotherapeutic agents to malign tissues or cells but also has a very important place in the optimization of complementary or alternative treatment solutions against the disease.

Anticancer drugs do have some negative effects such as healthy tissue toxicity, low stability, and drug resistance of tumor cells. New nanostructured drug delivery systems, using materials such as liposomes, polymeric drug conjugates, polymeric micelles, and solid lipid nanoparticles, can overcome these challenges. Importantly, these nanosized carrier materials improve the balance of efficacy and toxicity in therapeutic interventions.^4−6^

Recently, the encapsulation of iron oxide nanoparticles, within a suitable coating for inorganic drug delivery systems, has been reported as beneficial due to the prevention of particle aggregation in vitro and in vivo as well as the possibility for loading therapeutic molecules. The external surface of magnetic nanoparticle matrices can contain hydrophilic polymeric chains, or targeting ligands, for passive and/or active targeting of cancer tissues/cells. The biocompatible and biodegradable coating materials used in these core/shell nanoparticle systems usually consist of a polymeric material or a lipid-based structure. In this case, coating the surface of magnetic nanoparticles with solid lipid structures has provided good biomedical results. These matrixes, such as solid lipid nanoparticles, are biocompatible, biodegradable, and well-tolerated materials and so have found promising applications in drug delivery.^7^

By loading the magnetic particles into the SLNs, the drug reaches the tumor area by directing the magnetic field. Thus, toxicity and drug dosages are reduced, and validity, reliability, and patient compliance are greatly improved. In many cases, lipophilic drugs with good compatibility with the lipid matrix are chosen for loading into SLNs.^5^

Several studies were carried out by many research groups. In one of them, Hsu and Su tried to produce a prototype applicable in the field of magnetic temperature control medicine, in order to control hyperthermia of lipid particles, by encapsulating a lipophilic drug and iron oxide particles into lipid nanoparticles.^8^ In another study, Grillone et al. loaded magnetic iron oxide nanoparticles with sorafenib, an anticancer drug, and encapsulated it in solid lipid nanoparticles. They tried to ensure that the particles were delivered to the targeted area by applying an external magnetic field, thus aiming to minimize the negative effects on healthy tissues in cancer treatment.^9^

Docetaxel (DTX), from the taxane class, is a semisynthetic lipophilic anticancer drug that represents a new class with a different mechanism of action. Therefore, DTX plays a critical role in the treatment of solid tumors and is also used in clinical trials against ovarian carcinoma and breast, lung, and head/neck cancers. However, despite the advantages over other taxane class drugs, DTX’s clinical use is still limited due to its low solubility and serious side effects, which include neutropenia, peripheral neuropathy, and hypersensitivity reactions.

In conclusion, among drug delivery systems, liposomes are one of the most promising nanocarriers among many Food and Drug Administration (FDA)-approved formulations for cancer therapy. For this reason, based on the clinical trials, it is thought that the combination of DTX and liposomes will be more beneficial to cancer treatment due to its high potential for drug solubility and drug encapsulation.^10^

It has been reported in peer-reviewed scientific literature that the practice of using radionuclide-labeled nanoparticles is the most effective method for designing and testing novel nanoparticular systems. It is thought that the addition of technetium complexes to this new class of carrier systems will play an important role in future radiopharmaceutical research. Technetium-99 m (^99m^Tc) is the preferred radionuclide for the radiolabeling of nanoparticles as well as radiopharmaceuticals, due to its favorable imaging properties and easy availability.^11^

By conducting research in vitro and in vivo, we successfully combined docetaxel-loaded, folate receptor-targeted, radiolabeled magnetic solid lipid nanoparticles with ^99m^Tc. This combination resulted in the achievement of our ultimate goal of developing a theranostic drug delivery system that can be simultaneously used for tumor imaging and therapy.

## Materials and Methods

### Synthesis of Fol-PEG-CHEMS

First, a folate-polyethylene glycol-cholesterol hemisuccinate (Fol-PEG-CHEMS) molecule was synthesized to target the drug delivery system to folate receptors. Fol-PEG-CHEMS was synthesized according to the method reported previously.^12,13^ For the synthesis of Fol-PEG-amine, folic acid (0.06 mol), N-hydroxysuccinnimide (NHS) (0.074 mol), and dicyclohexylcarbodiimide (DCC) (0.048 mol) were dissolved in a mixture of tetrahydrofuran (THF) and dimethylsulfoxide (DMSO). Then, triethylamine (TEA) (0.025 mmol) and PEG-bisamine (0.05 mol) were added into the solution. The solution was allowed to stir overnight. Then, a Sephadex G-25 gel column was used to purify the product. For the synthesis of CHEMS-NHS, cholesteryl hemisuccinate (CHEMS) (109 mg), N-hydroxysuccinimide (NHS) (52 mg), and dicyclohexylcarbodiimide (DCC) (135 mg) were dissolved in dry THF and stirred at 0 °C for 1 h and then overnight at room temperature. The solvent was dried under vacuum, and the product was obtained. The obtained CHEMS-NHS was kept in 100% ethyl acetate overnight at +4 °C and purified by the recrystallization method and dried under vacuum. In the last step, Fol-PEG-amine (65 mg) and CHEMS-NHS (14 mg) were dissolved in 15 mL of chloroform, and the reaction was carried out overnight at room temperature. At the end of the reaction time, chloroform was removed using an evaporator, and the structure analysis of the synthesized Fol-PEG-CHEMS was performed by ^1^H NMR.

### Synthesis of Solid Lipid Magnetic Nanoparticles

For the synthesis of solid lipid magnetic nanoparticles, first of all, magnetic nanoparticles were synthesized using FeCl_2_·4H_2_O and FeCl_3_·6H_2_O as starting materials according to the method reported previously.^14^

Solid lipid magnetic nanoparticles were synthesized according to the modified method in the literature.^7,12^ Accordingly, folate receptor-targeted magnetic solid lipid nanoparticles (SLMNPs) were obtained using the emulsification method.^8^

Surface modification was performed by adding Fol-PEG-CHEMS nanoparticles to the lipid–water mixture. 40 mg of stearic acid was dispersed in 12 μL of oleic acid, 4 mL of acetone, and 4 mL of methanol in an ultrasonic bath for 10 min. Thereafter, 10 mg of Fol-PEG-CHEMS and the prepared lipid mixture were dispersed in 70 mL of distilled water at 70 °C, and MNPs (40 mg) were added to this mixture. The mixture was homogenized at 70 °C for 15 min, and Fol-PEG-CHEMS-modified SLMNPs were synthesized. The obtained magnetic solid lipid nanoparticles were lyophilized. For the stability of the lyophilized nanoparticles, the samples were stored in an environmental simulation chamber at 40 ± 2 °C/75% RH ± 5% RH for 4 months.

### Drug Loading of SLMNPs

20 mg of the obtained folate-conjugated solid lipid magnetic nanoparticles was dispersed in 4 mL of distilled water, and 5 mg/mL of docetaxel was added to the nanoparticles and kept in a homogenizer for 10 min at room temperature. Free docetaxel was removed using an Amicon ultracentrifugal filter. Drug entrapment and drug-loading efficiencies for docetaxel-loaded SLMNPs (DTX-SLMNPs) were determined by High performance liquid chromatography (HPLC).

### Characterization of SLMNPs

Hydrodynamic particle size analysis of DTX-SLMNPs was performed with a MALVERN ZETASIZER NANO ZS model DLS system. The prepared samples were dispersed in 1 mg/mL water, and the particle size and zeta potential measurements were performed.

In order to determine the morphology and particle size of MNPs, SLNs, and SLMNPs, Transmission electron microscopy (TEM) images were obtained with a JEOL-JEM 2100F model transmission electron microscope with a FEG electron gun operating under an accelerating voltage in the range of 80–200 kV.

The X-ray diffraction (XRD) patterns of MNPs and SLMNPs were analyzed by Thermo Scientific ARL K-alpha X-ray diffraction.

The magnetic properties of MNPs and SLMNPs were determined with a Dexing Magnet VSM 550 system. The magnetic moment of each dry sample was measured by applying a magnetic field between −3000 and +3000 Gauss. Then, the magnetic moment (emu) values obtained against Gauss were divided by the sample amount, and emu/g values were found. By drawing Gaussian versus emu/g graphs, the point at which the magnetic particle reaches magnetic saturation is determined in emu/g.

### Determination of the Encapsulation Efficiency

HPLC method was used to determine the amount of drug entrapped in the synthesized SLMNPs. Docetaxel samples were prepared at concentrations of 500, 250, 125, 62.25, and 31.12 μg/mL to determine the drug entrapment capacity. The calibration graph was drawn with the areas found as a result of the calculations. In order to determine the drug content of the synthesized SLMNPs, 1 mg of DTX-SLMNP was dissolved in 1 mL of methanol and then kept in an ultrasonic bath for 10 min, and HLPC analysis was performed. In the HPLC method, water (solvent A) and acetonitrile (solvent B) were used as mobile phases. The mobile flow system was programmed with a gradient pattern where the composition ratios varied over time. In the first 15 min, it consisted of 65% A and 35% B. From 15 to 25 min, the ratio changed to 35% A and 65% B. Then, from 25 to 30 min, it became 25% A and 75% B. At 30 to 35 min, the ratio shifted to 5% A and 95% B. After 35 min, the system was set to a constant gradient of 100% B per minute. Additionally, the UV detector's wavelength was adjusted to 230 nm, the temperature was maintained at 25 °C, and the flow rate was set to 1 mL/min..

### Determination of Drug Release

The drug release of docetaxel-loaded magnetic solid lipid nanoparticles was determined in vitro at 37 °C using a dialysis membrane. For this purpose, 4 mg of magnetic solid lipid nanoparticles was dispersed in 2 mL of PBS (pH 7.4) in an ultrasonic bath and added to a dialysis membrane and placed in 25 mL of PBS (pH 7.4) buffer in a beaker. 1 mL of the sample was taken from the PBS buffer at certain time intervals (30 min, 1 h, 2 h, 3 h, 4 h, 5 h, and 6 h) during 6 h, and 1 mL fresh buffer was added instead. The amount of docetaxel was determined by HPLC.

### Radiolabeling Studies

Labeling studies of docetaxel-loaded solid lipid magnetic and docetaxel-free solid lipid magnetic nanoparticles with ^99m^Tc were performed using the SnCl_2_ reduction method. Nanoparticles loaded with 500 mg of docetaxel were dispersed in 100 μL of methanol, and 25 μg/μL of SnCl_2_ solution and 250 μCi ^99m^Tc were added and incubated at 80 °C for 1 h (^99m^Tc-DTX-SLMNP). The radiochemical purity of the radiolabeled compound was determined by precipitating it with a magnet due to its magnetic property and counting the radioactivity of the supernatant and the precipitate phase in a dose calibrator.

### *In Vitro* Studies

Folate receptor-positive SKOV-3, ovarian serous cystadenocarcinoma, and folate receptor-negative A549 lung (carcinoma) epithelium were used to examine the cellular level activities of ^99m^Tc-labeled DTX-loaded folate receptor-targeted solid lipid magnetic nanoparticles (^99m^Tc-DTX-SLMNPs).

### Cytotoxicity Study

Cytotoxicity studies were performed on SKOV-3 and A549 cell lines using different concentrations of MNP, SLMNP, DTX, and DTX-SLMNP compounds (2 mM, 1 mM, 500 μM, 100 μM, 50 μM, and 10 μM).

IC_50_ (the concentration range causing 50% mortality) values were determined by MTT test (2-(2-methoxy-4-nitrophenyl)-3-(4-nitrophenyl)-5-(2,4-disulfophenyl)-dihydroxy tetrazolium) colorimetrically. Cell suspensions were prepared at 10^5^ cells/mL/well in 96-well microplates. 100 μL of cell suspension was added to each well, and DTX, SLN-MNP-DTX, MNP, and SLN-MNP samples at the five different concentrations mentioned above were added to the wells except the control. Each parameter was studied by repeating five times.

Cells were incubated at 37 °C in 5% CO_2_ for up to 48 h. At the end of the incubation, 10 μL of MTT solution was added to each well, and after 4 h of incubation, the absorbance value (OD) of each well was read using a spectrophotometer at 450 nm wavelength and 690 nm reference ranges. The % cytotoxicity values were calculated using the formula given below.

%cytotoxicity = 1 – (measured optical density value/control value) × 100

### Incorporation Studies

Incorporation studies of ^99m^Tc-labeled SLMNP and MNP samples were performed on SKOV-3 and A549 cells.

1st group DTX-SLMNPs labeled with ^99m^Tc: for each cell line, 50 μCi ^99m^Tc and radiolabeled 10 μg nanoparticles were added to each of the 12 wells in 500 μL medium.

2nd group DTX-SLMNPs labeled with ^99m^Tc applied under the magnetic field: for each cell line, 50 μCi ^99m^Tc and 10 μg radiolabeled nanoparticles were added to each of the 12 wells in 500 μL medium. Unlike the 1st group, a magnetic field was applied to the cell plates with an external magnet during the incubation period.

3rd group MNPs labeled with ^99m^Tc: for each cell line, 50 μCi ^99m^Tc and 10 μg of radiolabeled nanoparticles were added to each of the 12 wells in 500 μL medium.

Cells were washed with 0.9% NaCl by discarding the radiolabeled media added to the flasks as described above on the cells at 30, 60, 120, and 240 min, and the remaining activity in the cells was counted with a Cd(Te) detector. As a result, the % binding efficiency per cell was calculated from the activity values per cell.

### Scintigraphic Images

Gamma camera imaging studies were performed on animal models with tumors. Animal studies were carried out by researchers having Experimental Animal Use Certificate in line with the permit number 77.634.435 obtained from Celal Bayar University Animal Experiments Local Ethics Committee on 22/08/2017 and 2022-015 obtained from Ege University Animal Experiments Local Ethics Committee.

The biodistribution of the radiolabeled molecule in the body on three healthy balb-c mice was determined by the images taken on a SPECT camera in order to decide on the tumor site before the tumor formation studies on mice. From the SPECT images taken from healthy mice, it was understood that ^99m^Tc-DTX-SLMNP uptake occurred in the liver and bladder. Therefore, the tumor formation site in nude mice was determined as the upper shoulder.

Tumor formation in nude CD-1 mice: Folate receptor-positive SKOV-3 and folate receptor-negative A549 cells were grown in a medium consisting of DMEM, 100 U/mL penicillin, 100 mg/mL streptomycin, and 0.3 mg/mL glutamine. Cells were injected subcutaneously (SQ) into nude CD-1 mice at 5 × 10^6^ cells in 200 μL of DMEM. SKOV-3 cells were injected from the left upper shoulder and A549 cells from the right upper shoulder as SQ. At the time of adaptive transfer, the tumor size was followed for 3 weeks. It was used in in vivo imaging studies when tumors reached a moderate size. Twelve female mice were used for the imaging study.

In this study, it is aimed to target the synthesized radiolabeled magnetic solid lipid nanoparticles to the tumor region with the effect of an externally applied magnetic field. For this reason, in the second group of mice, 1.17–1.21 Tesla NdFeB magnets were applied to the tumor area after the *i.v.* injection of the radiolabeled substance, and the magnetic field was applied during the entire scintigraphic procedure.^15^

In the first group, 3.7 MBq/0.1 mL (0.1 mCi/0.1 mL) of the ^99m^Tc-DTX-SLMNP compound was administered intravenously from the tail vein of nude CD-1 mice.

In the second group, 3.7 MBq/0.1 mL (0.1 mCi/0.1 mL) of the ^99m^Tc-DTX-SLMNP compound was administered intravenously from the tail vein of nude CD-1 mice. The magnetic field was applied during the entire scintigraphic procedure to the tumor regions of the mice from the outside.

Ketamine+xylazine (75–100 mg/kg + 5–10 mg/kg) anesthesia was administered intraperitoneally (i.p.) to each mouse before the injection in order to relieve the anxiety of the mice and prevent them from feeling the pain. Static images were taken 15 min, 60 min, 120 min, 240 min, and 24 h after the injection and repeated. For the static images, a 1024 × 1024 matrix, 2-zoom, low-energy high-resolution collimator was used.

## Results and Discussion

### Synthesis of Fol-PEG-CHEMS

**Figure 1:**
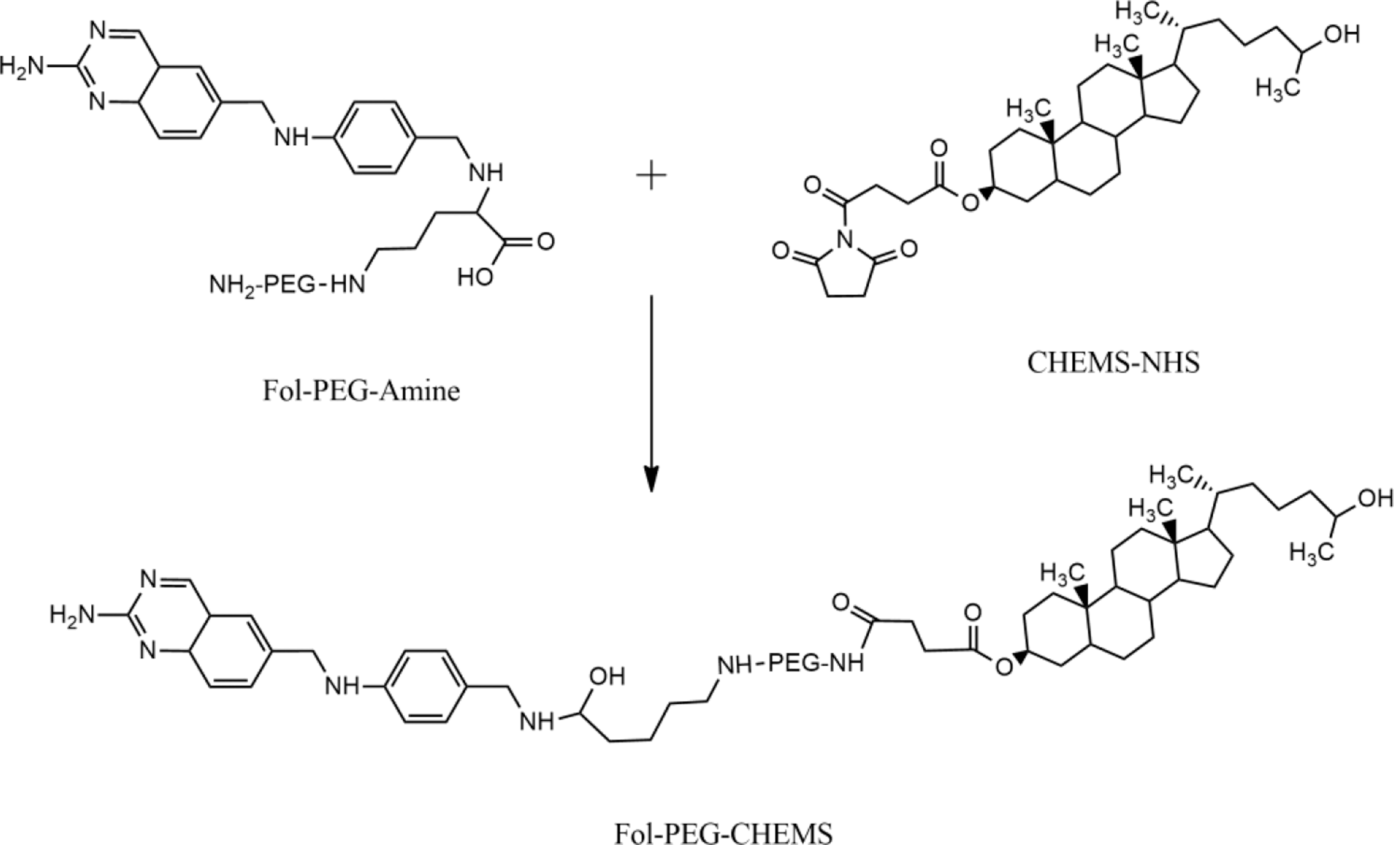
Schematic illustration of the reaction steps for FOL-PEG-CHEMS synthesis.

Synthesis steps for Fol-PEG-CHEMS were carried out in accordance with the literature. First, the CHEMS-NHS was obtained, and then Fol-PEG-amine was synthesized. Fol-PEG-CHEMS was prepared using these two products (Figure 1). ^1^H NMR analysis was also performed for the final product Fol-PEG-CHEMS. The related peaks were observed for the folate moiety––8.65(d), 7.63(d), 6.63(d), 4.49(d), and 4.26(m); the PEG moiety––3.77(m), and the CHEMS moiety––5.31(bd) and 2.26(m). However, the ^1^H NMR peak shows the characteristic peaks of similar functional groups, confirming the successful synthesis of Fol-PEG-CHEMS molecules in the previously reported data.^3,12^

### Preparation and Characterization of Magnetic Solid Lipid Nanoparticles

According to the modified method, folate receptor-targeted magnetic solid lipid nanoparticles (SLMNPs) were obtained using the emulsification method.^7,8,12^

In our study, the zeta potential values of MNP, SLMNP, and DTX-SLMNP were determined as −21.3, −34.4, and −20.8 mV, respectively, indicating the stability of the particles in solution. As a result, it can be concluded that the coating of magnetic particles with a lipid layer prevents the formation of aggregates.

**Figure 2:**
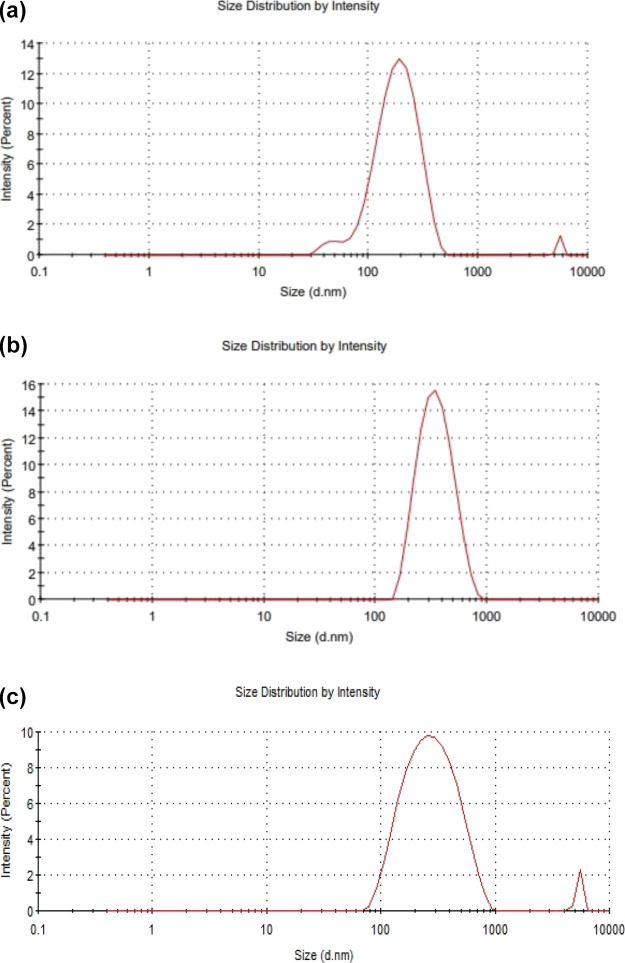
Nanoparticle size distribution of (a) MNPs, (b) SLMNPs, and (c) DTX-SLMNPs in aqueous media.

According to the intensity versus size graph given in Figure 2a, the mean hydrodynamic diameter of the magnetic nanoparticles that dispersed in water was 189.1 nm with a polydispersity index (PDI) of 0.327, showing a good distribution.^14^ The mean hydrodynamic diameter of the SLMNPs dispersed in water was found to be 328.6 nm, and the polydispersity index was 0.145 nm (Figure 2b). These values show that the hydrodynamic diameter of the nanoparticles increases because of the modification of the surface of the nanoparticles with a lipid layer. When docetaxel was loaded on SLMNPs, the mean hydrodynamic diameter of the particles in water was found to be 317 nm, and the polydispersity index was 0.480 (Figure 2c). Docetaxel-loaded nanoparticles have no significant difference in the particle size because of drug loading (Table 1). According to the results of DLS analysis performed after the SLMNPs were stored at 40 °C/75% RH for 4 months, no significant difference was observed in the particle size and zeta potential. This result shows that the SLMNPs are stable in terms of particle size at 40 °C for a duration of 4 months.

**Table 1:** DLS Measurements of MNP, SLMNP, and DTX-SLMNP

particles	size (nm)	PDI	zeta potential (mV)
MNP	189.1	0.327	–21.3
SLMNP	328.6	0.145	–34.4
DTX-SLMNP	317	0.480	–20.8

**Figure 3:**
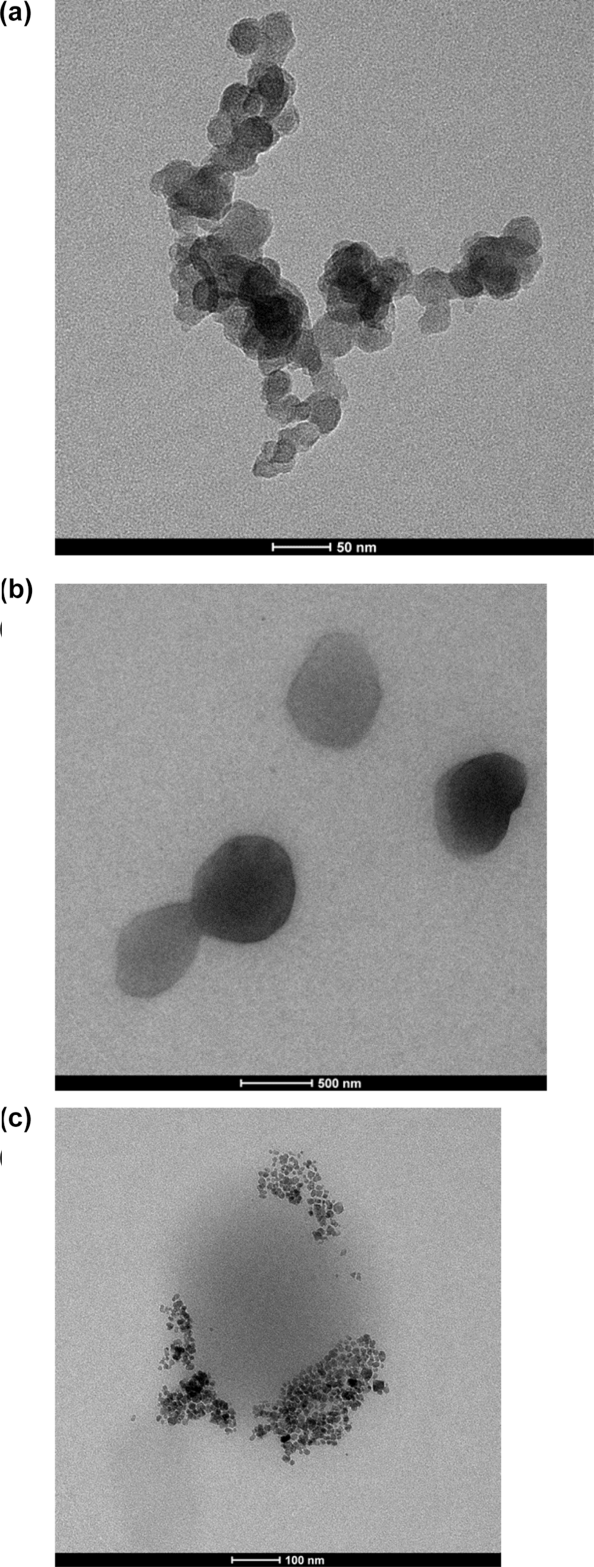
TEM images of (a) MNP, (b) SLN, and (c) SLMNP.

The TEM images of MNPs, SLNs, and SLMNPs are shown in Figure 3. Iron nanoparticles, having magnetic properties, come together in a solution environment and aggregate; thus, it is difficult to use the particles for their intended purpose. For this reason, it is necessary to coat the particle surface with a coating agent that will prevent the particles from agglomeration. In this study, magnetic nanoparticles were embedded in solid lipid nanoparticles for this purpose. According to the TEM images, the size of the MNPs was observed between 25 and 35 nm, SLNs between 400 and 500 nm, and SLMNPs between 350 and 450 nm. It was observed that when iron nanoparticles were loaded into solid lipid nanoparticles, a reduction in lipid nanoparticle size was observed. Similar to this result, Zhao et al. reported that the size of the lipid nanoparticles decreased after the magnetic nanoparticles were embedded.^5^

**Figure 4:**
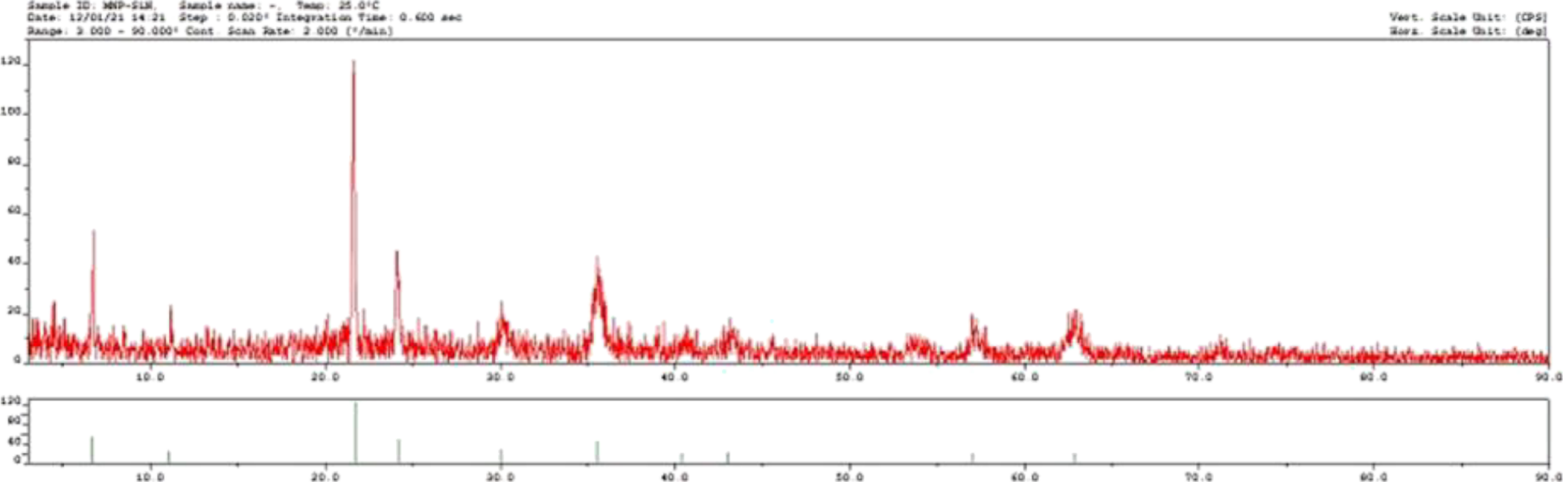
XRD patterns of SLMNPs.

The crystal structures of MNPs and SLMNPs were analyzed by XRD (Figure 4). The diffraction peaks of MNPs (220), (311), (400), (422), (511), and (440) were matched to the reference magnetic crystal peaks, which all agree with the literature data. The fact that the diffraction peaks are not very sharp indicates that the synthesized magnetic nanoparticles have an amorphous and fine crystal structure. It is understood that the magnetic nanoparticles synthesized from the obtained diffraction peaks match with the reference crystal magnetite (Fe_3_O_4_) peaks.^15^ The magnetic properties of MNPs and SLMNPs were analyzed by VSM. The saturation of MNPs was found to be equal to 63.77 emu/g, while that of the lipid-coated SLMNPs was 28.49 emu/g. Considering the VSM results of MNP and SLMNP, it was concluded that coating of the surface such as polymer, amino-silane, and so forth caused a decrease in the magnetic saturation of the particles.^15,16^

### Determination of the Encapsulation Efficiency

HPLC analysis to understand the DTX loading efficiency on magnetic solid lipid nanoparticles was performed, as described in the Materials and Methods section. The retention time (*R*_t_) of DTX was observed as 17.23 min in the chromatogram. The calibration curve was plotted for each concentration. The entrapment efficiency of drug-loaded DTX-SLMNs was calculated from the formula given below and was found to be 19%.

Ee = ((Wa – Ws)/Wa) × % 100Ee: encapsulated drug entrapment efficiency; Wa: amount of drug encapsulated in NPs; Ws: amount of drug that remained in the supernatant.

### Determination of Drug Release

The drug release efficiency of the synthesized docetaxel-loaded solid lipid nanoparticles was determined at pH = 7.4 and 37 °C using a dialysis membrane in vitro. According to the results obtained, while the drug release value was 5.7% in the first 30 min, it was 12.8% at the end of the 6th hour.

### Radiolabeling Studies

^99m^Tc is a suitable radionuclide for gamma imaging to measure the biodistribution of nanoparticles quantitatively and also to visualize the whole-body distribution. Thus, we use the direct labeling of DTX-SLMNPs with ^99m^Tc using stannous chloride as the reducing agent. The radiolabeling yield of SLMNPs was found to be 98.0 ± 2.0% (*n* = 6).

### Biological Behavior of Solid Lipid Magnetic Nanoparticles

#### In Vitro Studies

In this study, all in vitro studies were performed using folate receptor-positive SKOV-3, ovarian serous cystadenocarcinoma, and folate receptor-negative A549 lung (carcinoma) epithelium. The IC_50_ values for DTX, DTX-SLMNP, and SLMNP were found to be 168.36, 172.27, and 172.6 μM at 48 h in the A549 cell line and 18.09, 50.1, and 115.72 μM for the SOV-3 cell line. It was determined that the IC_50_ value of DTX-SLMNP in folate receptor-positive SKOV-3 cells was lower than that of the A549 cell line. As a result, it was understood that the designed biocompatible system was specific for the folate receptor in accordance with its purpose. In addition, considering the data obtained in the MTT test, the high IC_50_ value of solid lipid magnets that are not loaded with docetaxel shows that the nanoparticles do not have a high toxic effect in the cell. Hami et al. synthesized pH-responsive docetaxel-conjugated poly(lactic acid) (PLA)-polyethylene glycol (PEG) micellar formulations. They reported that the IC_50_ value of DTX on the SKOV-3 cell line was 8.42 ± 1.61 ng/mL at 72 h, which is comparable with our findings.^17^

### Time-Dependent Incorporation Studies

**Figure 5:**
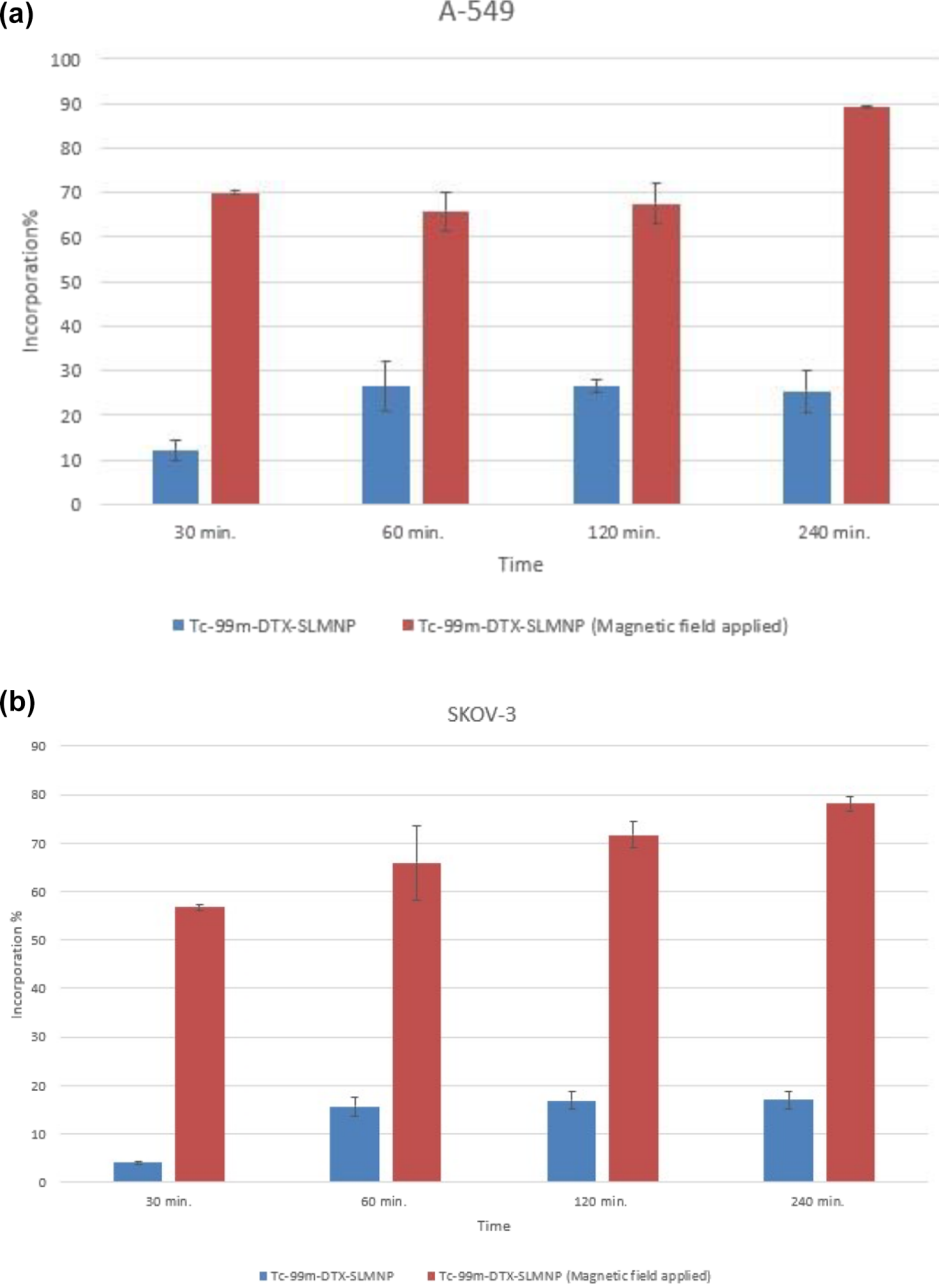
Incorporation of ^99m^Tc-DTX-SLMNPs and ^99m^Tc-DTX-SLMNPs (magnetic field-applied) vs time on (a) A549 and (b) SKOV-3 cell lines.

Time-dependent incorporation studies for ^99m^Tc-DTX-SLMNP and ^99m^Tc-DTX-SLMNP (magnetic field-applied) compounds on A549 and SKOV-3 cells were performed. The radiolabeled compounds were incubated with cells in culture media at distinct time points. The medium on the cells was discarded and washed with 0.9% NaCl to examine the time parameter. The remaining activity in the cells was counted with a Cd(Te) detector, and the binding efficiencies were calculated from the activity values per cell over time. The percent binding efficiencies of ^99m^Tc-DTX-SLMNP and ^99m^Tc- DTX-SLMNP (magnetic field-applied) compounds on A549 and SKOV-3 cells are given in Figure 5, respectively.

According to the results obtained from A549 and SKOV-3 cell lines, the highest uptake efficiencies of A549 and SKOV-3 cells were observed as (25.46 ± 4.76%, 17.04 ± 1.87 for ^99m^Tc-DTX-SLMNP and (89.29 ± 0.17%, 78.10 ± 1.38) for ^99m^Tc- DTX-SLMNP (magnetic field-applied) at 240 min.

The ^99m^Tc- DTX-SLMNP (magnetic field applied) showed a higher binding than the ^99m^Tc-DTX-SLMNP from the uptake efficiency of radiolabeled nanoparticles in the cell. It is understood that the application of magnetic field on magnetic nanoparticles increases the cell uptake efficiency. The uptake yield of both radiolabeled compounds was gradually increased with time for both cell lines.

#### *In Vivo* Studies

**Figure 6:**
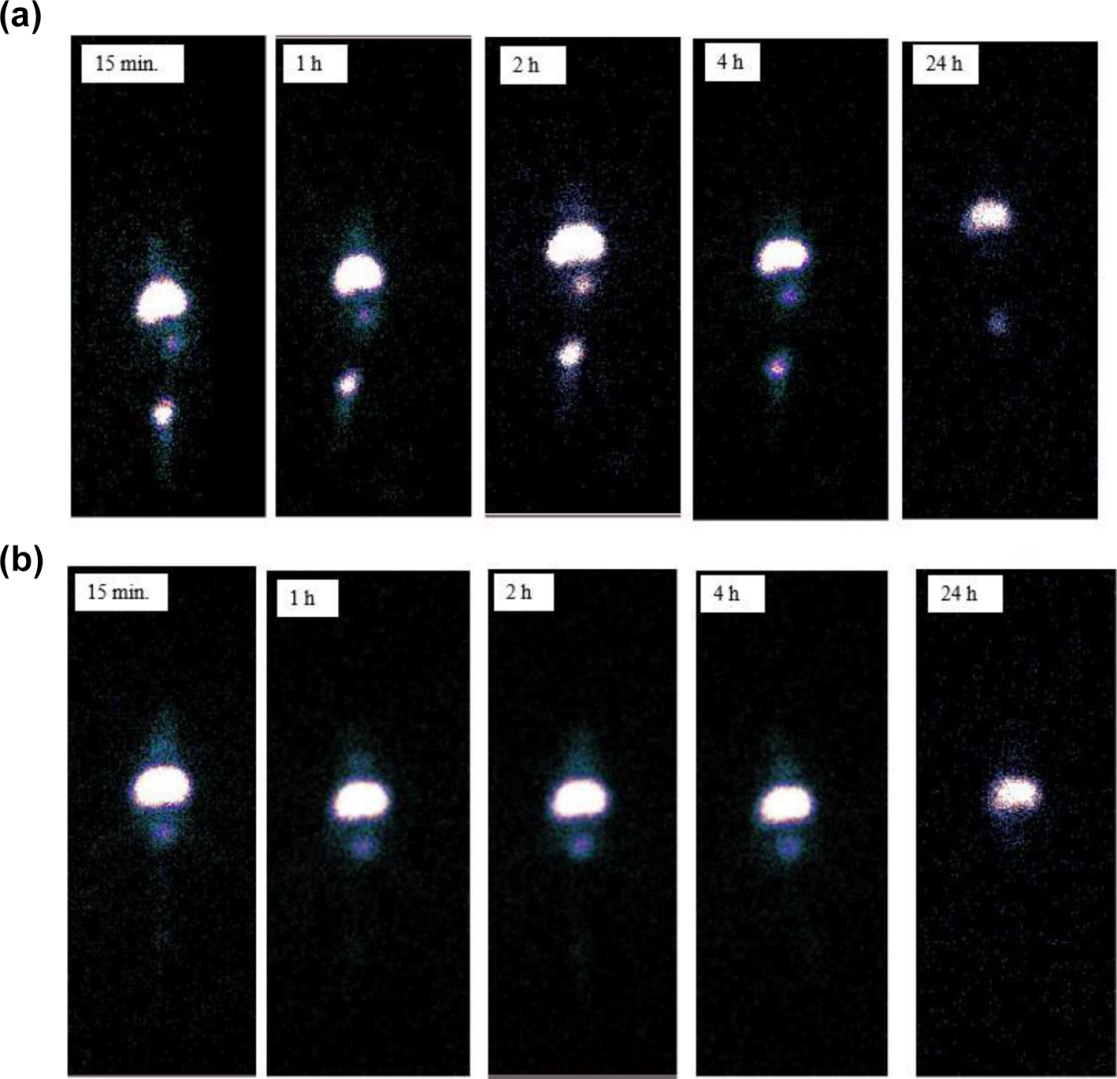
Scintigraphic images of (a) ^99m^Tc-DTX-SLMNPs and (b) ^99m^Tc-DTX-SLMNPs (applied magnetic field on tumor regions) at 15 min, 1 h, 2 h, 4 h, and 24 h.

The surface of radiolabeled magnetic solid lipid nanoparticles synthesized in this study was modified with folic acid to be specific for folate receptors. In addition, it is expected that the uptake of DTX-SLMNPs in the tumor region will increase with the effect of an external magnetic field, thanks to the magnetic nanoparticles in the solid lipid nanoparticles. For this reason, while no magnetic field was applied to the first group of nude mice, a magnetic field was applied to the tumor area with 1.17–1.21 Tesla NdFeB magnets during the entire scintigraphic attraction period after the *i.v.* injection of the radiolabeled substance in the second group of nude mice. After the *i.v.* injection of ^99m^Tc-DTX-SLMNPs in the first and second group nude mice, SPECT images were taken at 15 min, 1 h, 2 h, 4 h, and 24 h and are shown in Figure 6a,b. These images show the uptake of ^99m^Tc-DTX-SLMNPs in the liver and bladder regions. Tumor uptake was evaluated according to the regions of interest (ROIs) in the images.

Accordingly, it was observed that the right tumor uptake of the radiolabeled molecule was found to be 2.32 ± 0.92 at the 15th min without the magnetic field and found to be 2.10 ± 0.77 when magnetic field was applied. Similarly, while the left tumor uptake was 3.35 ± 1.37 at the 15th min, it was calculated as 3.13 ± 1.86 when magnetic field was applied. According to these results, magnetic field did not have an effect on tumor uptake due to the short duration of magnetic field application in the first 15 min. However, after the 1st hour, it was observed that the uptake in both the right and left tumor regions increased over time and with the application of magnetic field. At the 2nd hour, it was understood that the uptake reached its highest value in the region of both the right tumor (without magnetic field application: 2.94 ± 1.52 and with magnetic field application: 3.90 ± 0.75) and left tumor (without magnetic field application: 3.72 ± 1.42 and with magnetic field application: 4.03 ± 0.17) (Figure 6b and Table 2). However, it was observed that the uptake in the tumor region formed by folate receptor-positive SKOV-3 cells at 1, 2, and 4 h was higher than the uptake in the tumor region formed by folate receptor-negative A549 cells. Thus, we conclude that folic acid on the surface of magnetic lipid nanoparticles in accordance with the purpose of the project directs the structure to folate receptors, and furthermore, the uptake in this region increases with the externally applied magnetic field. Worth to be mentioned is the work of Zhao et al., where cisplatin was loaded into magnetic lipid nanoparticles, and the targeting ability of the drug delivery system, due to the presence of the magnetic field, was demonstrated. They concluded that this magnetic lipid carrier system can transport cisplatin to the target area under an external magnetic field as a result of in vivo studies.^5^ İçhedef et al. investigated the magnetic field effect on the biodistribution of radiolabeled magnetic nanoparticles in rabbits and concluded that the nanoparticle uptake in the targeted region was higher than that without the magnet.^15^

**Table 2:** ROI Values of the Uptake of ^99m^Tc-DTX-SLMNPs in Tumor-Bearing Nude CD-1 Mice

organs	applied magnetic field	ROI values			
		15 min	1 h	2 h	4 h
tumor folate-negative	no	2.32 ± 0.92	1.70 ± 0.46	2.94 ± 1.52	2.76 ± 1.15
	yes	2.10 ± 0.77	2.64 ± 1.02	3.90 ± 0.75	3.48 ± 1.55
tumor folate-positive	no	3.35 ± 1.37	2.06 ± 0.61	3.72 ± 1.42	3.27 ± 0.81
	yes	3.13 ± 1.86	3.29 ± 1.87	4.03 ± 0.17	3.99 ± 1.20
right lung	no	5.93 ± 2.05	3.28 ± 0.91	7.79 ± 3.20	8.15 ± 4.13
	yes	6.23 ± 1.18	8.57 ± 1.09	8.69 ± 4.74	8.11 ± 1.51
left lung	no	5.23 ± 1.46	3.54 ± 0.56	6.93 ± 2.19	6.80 ± 2.88
	yes	5.89 ± 0.69	7.83 ± 1.88	8.13 ± 2.46	7.15 ± 1.49
liver	no	16.67 ± 5.97	8.85 ± 4.87	12.14 ± 3.28	30.05 ± 1.84
	yes	58.10 ± 10.26	235.42 ± 49.22	242.67 ± 85.86	258.69 ± 21.11

The metabolic clearance of nanoparticles occurs primarily through two pathways: (1) urinary excretion and (2) hepatobiliary and feces excretion. When the size of nanoparticles exceeds 6 nm or when they contain heavy metals, they are efficiently captured by the reticuloendothelial system (RES) in the liver and spleen. Conversely, nanoparticles with small sizes (<5.5 nm) are rapidly eliminated through the urinary system as they are small enough to pass through the kidney filtration threshold.^18,19^ Thus, SLMNPs would excreted by hepatobiliary and feces excretion.

Since the folic acid receptor (FR) is expressed in many human cancers, including ovarian, brain, kidney, and lung malignancies, it is used as an important target in the design of tumor-specific drug delivery systems. Folic acid is a widely used molecule to target metastatic tumors due to its high affinity for the folate receptor. The use of radiolabeled folate-conjugated imaging agents is the most effective method to determine whether folate-conjugated systems accumulate in tumor cells or not.^10^ Thus, in the current study, we used radiolabeled folate-conjugated magnetic lipid nanoparticles to assess the tumor accumulation on folate receptor-positive SKOV-3 cells. SPECT imaging results showed that ^99m^Tc-DTX-SLMNPs were accumulated on SKOV-3-mediated tumors more than on the A549 cell-mediated tumor, which proves our purpose.

## Conclusions

In the current study, a radiolabeled-folate-mediated solid lipid magnetic nanoparticle (SLMNP) system has been successfully formulated. It was developed by loading superparamagnetic iron oxide nanoparticles (MNPs) and docetaxel (DTX) into the solid lipid nanoparticles. The loading of magnetic nanoparticles into the solid lipid nanoparticles prevents the aggregation of magnetic nanoparticles in the solution and also reduces the size of the solid lipid nanoparticles. The resulting magnetic SLNs showed an obvious magnetic targeting effect *in vivo*.

The results of these tests demonstrate that ^99m^Tc-DTX-SLMNP accumulates on SKOV-3-mediated tumors more than on A549 cell line-mediated tumors. Thus, the obtained data support our original assumption. Our conclusion is that folic acid on the surface of magnetic lipid nanoparticles, in accordance with the purpose of the study, directs the structure to folate receptors and that the uptake in this region increases with an externally applied magnetic field.

In this multidisciplinary study, a theranostic system, that is, a drug delivery system that enables therapy and imaging to be performed simultaneously, has been created for the first time.
